# Profile and Response to Anti-Tuberculosis Treatment among Elderly Tuberculosis Patients Treated under the TB Control Programme in South India

**DOI:** 10.1371/journal.pone.0088045

**Published:** 2014-03-11

**Authors:** Banu Rekha Vaithilingam Velayutham, Dina Nair, Vedachalam Chandrasekaran, Balambal Raman, Gomathy Sekar, Basilea Watson, Niruparani Charles, Muniyandi Malaisamy, Aleyamma Thomas, Soumya Swaminathan

**Affiliations:** National Institute for Research in Tuberculosis (formerly Tuberculosis Research Centre), Chennai, India; San Francisco General Hospital, University of California San Francisco, United States of America

## Abstract

**Introduction:**

The demographic transition in India has resulted in an increase in the elderly population. There is limited data on the profile of elderly tuberculosis (TB) patients and their treatment outcomes in India.

**Objective:**

To compare the clinical profile, presentation and response to anti-TB treatment among elderly (≥60 yrs) and younger (15–59 yrs) TB patients treated under the Revised National TB Control programme.

**Methodology:**

Retrospective cohort analysis of TB patients treated from May 1999 to December 2004 in one Tuberculosis Unit of Tiruvallur district, South India.

**Results:**

Records of 865 elderly and 4343 younger TB patients were examined: elderly were more likely to be male (84% vs. 71%), smokers (46% vs.37%), illiterate (63% vs. 45%), identified by active case finding through survey (19% vs. 11%), have pulmonary TB (96% vs. 91%) and initial smear negative disease (46% vs. 36%) compared to younger (for all p<0.001). Among a total of 352 elderly and 1933 younger new smear positive pulmonary TB, the elderly had higher loss to follow-up (15% vs. 11%; p = 0.03) and death rates (9% vs. 4%; p<0.001). *Mycobacterium tuberculosis* susceptibility to first line anti-TB drugs did not differ (elderly 87% vs. younger 84%) (p = 0.20). Side effects related to anti-TB drugs were reported by a higher proportion of elderly patients (63% vs. 54%) (p = 0.005). Previously treated patients had similar treatment outcomes in both the groups.

**Conclusion:**

Elderly TB patients are less likely to have smear positive disease. Newly diagnosed elderly TB patients are more likely to be lost to follow-up or die and report drug side effects. Suitable interventions need to be developed for effective management and better treatment outcomes of TB in the elderly.

## Introduction

The demographic transition in India has resulted in the label of an ageing nation [1]. The Government of India's ‘National Policy on Older Persons’ defines ‘elderly’ as a person who is of age 60 years or above [2]. In 2001, the proportion of elderly in India was about 7.4% of the overall population which increased to 8.3% in 2011 [2], [3]. A decline in immunity and age related physiological changes leads to an increased burden of communicable and non-communicable diseases in the elderly [1].

India is the highest tuberculosis (TB) burden country accounting for a quarter (26%) of the global incidence [4]. About 90% of TB in the elderly is due to endogenous reactivation [5]. Co-morbidities, inter-current illness, malnutrition, excess alcohol use, underlying malignancy and the use of immunosuppressant drugs can impair the cell-mediated immunity in the elderly causing progression to TB disease [6]. New smear positive TB case notification in India in those aged 55 yrs and above showed an increase from 31133 to 132938 between the years 2000 to 2012 [4]. This increase in TB case notifications may partly be due to the increase in the elderly population and is expected to rise further since the proportion of elderly (≥60 yrs) in India projected for 2026 is about 12.17% of the overall population [1]. A study from South India has documented that older TB patients (≥60 yrs) accounted for 14% of all TB patients [7]. There is limited data on the profile of elderly TB patients and their treatment outcomes in India [7]–[12]. The objective of this study was to compare the clinical profile, presentation and response to anti-TB treatment among the elderly (≥60 yrs) and younger (15–59 yrs) TB patients treated under the Revised National TB Control programme (RNTCP).

## Methodology

### Study area and participants

This was a retrospective analysis of a cohort of TB patients registered for anti-TB treatment from May 1999 to December 2004 in one Tuberculosis Unit (TU) of Tiruvallur District, Tamil Nadu State, South India [Under the RNTCP, a TU is a sub-district supervisory unit which covers on average a population of 500,000]. This TU has 17 government health care facilities including seven designated microscopy centres.

National Institute for Research in TB (NIRT) (formerly Tuberculosis Research Centre) was monitoring the TB programme implemented by the Government of Tamil Nadu in one TU of Tiruvallur district, since May 1999. TB cases were diagnosed by testing symptomatic patients at health facilities through the examination of three sputum smears for acid-fast bacilli (passive screening). In addition, the centre was conducting epidemiological surveys from 1999 to measure the prevalence of TB (active screening) in this TU. Approval of the Ethics Committee of NIRT was obtained and written informed consent was obtained from all the patients (aged≥18 yrs) and from guardian/care-givers for those aged 15 to less than 18 yrs.

All patients diagnosed with TB were given directly observed treatment in accordance with the RNTCP guidelines with first line anti-TB drugs which include isoniazid (H), rifampicin (R), pyrazinamide (Z), ethambutol (E) and streptomycin (S) [13]. Newly diagnosed TB patients received Category I (2H_3_R_3_Z_3_E_3_/4H_3_R_3_) or Category III (2H_3_R_3_Z_3_/4H_3_R_3_) anti-TB regimens, while previously treated TB patients received Category II regimen (2H_3_R_3_Z_3_E_3_S_3_/1H_3_R_3_Z_3_E_3_/5H_3_R_3_E_3_) [13]. The treatment was extended for one month if the smears were positive at the end of the intensive phase (IP) in Categories I and II. Every dose of treatment in IP, and at least the first of the three doses during the continuation phase were given under supervision. Details of patients who began treatment were noted in the register, and they were monitored in accordance with the RNTCP guidelines [13].

### Data collection

The information about type of disease (pulmonary or extra-pulmonary), category of disease, grade of smear and baseline weight (in kg) was obtained from the treatment cards. Two sputum samples were collected from each patient for smear and culture on Lowenstein Jensen (L-J) medium, within a week of treatment initiation. The specimens that yielded growth were tested for drug susceptibility. Resistance to H and R was determined by the absolute concentration method minimum inhibitory concentration (MIC) and to S by resistance ratio (RR) method [14], [15]. MICs of ≥5 mg/l and of ≥128 mg/l were defined as resistance to H and R, respectively. A RR of ≥8 was considered as resistance to S [16].

Within 8 weeks from the start of treatment, trained medical social workers (MSW) interviewed patients using a structured questionnaire to obtain information about education, co-morbid conditions, personal habits, chest symptoms, care-seeking behaviour and drug related problems. MSW's made three attempts to contact each patient for the interview.

Upon completion of treatment, each patient's treatment card was again reviewed to collect information about follow-up sputum smear results and treatment outcomes.

### Case definitions

Patients aged ≥60 yrs were defined as elderly and those aged 15–59 years as younger. RNTCP definitions were used to classify the type of TB, category and treatment outcomes [13].

Patients who could read and write were considered literate; those who said they habitually consumed alcohol or smoked as alcohol users or smokers respectively, patients on treatment with hypoglycaemics were considered diabetics.

Multidrug resistance (MDR-TB) was defined as resistance to both H and R, with or without resistance to other drugs.

### Study outcome

The younger and elderly group were compared by gender, body weight in Kg, literacy rate, employment status, diabetes, asthma, smoking, alcohol use, respiratory symptoms and its duration, type of TB, sputum smear status, side effects and drug susceptibility profile. TB treatment outcomes were compared as; those cured, treatment completed, failed, loss to follow-up (referred to as ‘default’ in RNTCP nomenclature), died and transferred out.

### Data entry and analysis

Data were double-verified, entered and analysed using SPSS version 14.0 (Statistical Package for the Social Sciences Inc, Chicago, IL, USA). Variables were expressed as proportions. Statistical differences between the elderly and younger groups were determined with chi-square test and Yates correction, except when expected values of less than 5 required the use of the Fisher exact test. Significance was determined at 5%. Crude odds ratios (OR) and 95% confidence intervals (CI) were determined using “younger” as the reference group.

## Results

### Patient characteristics

From May 1999 through December 2004, 5368 TB patients were registered for treatment. Patients aged <15 yrs (n = 65) and those on non- supervised TB treatment (n = 95) were excluded. Among the remaining 5208 patients included in the analysis, there were 865 elderly (60–90 yrs) and 4343 younger (15–59 yrs) TB patients.

The median age in the elderly group was 65 years (IQR: 60–68) and younger group was 39 years (IQR: 28–49). The baseline characteristics of elderly and younger patients are shown in Table 1. The socio-demographic profile of elderly showed a male preponderance (84% vs. 71%; p<0.001), higher illiteracy (63% vs. 45%; p<0.001) and unemployment (58% vs. 35%; p<0.001) (Table 1). The mean body weight was similar in both the groups; elderly 41 kg (range 24–77 kg) and younger 42 kg (range 21–94 kg).The proportion of patients who smoked was higher in the elderly (46% vs. 37%; p<0.001) while the consumption of alcohol was not different (29% and 30%). Prevalence of reported diabetes mellitus did not differ in both the groups (6% vs. 5%).

**Table 1:** Baseline characteristics of TB patients in the elderly and younger group.

Characteristics	Elderly n = 865		Younger n = 4343		OR (95% CI)$	P value
	n	%	n	%		
**Male**	723	*84*	3083	*71*	2.08 (1.72–2.52)	<0.001
**Body wt** * ≤40 Kg	385	*48*	1838	*45*	1.1(0.96–1.31)	0.13
**Illiterate** **	541	*63*	1941	*45*	2.06 (1.78–2.39)	<0.001
**Un-employed**	498	*58*	1506	*35*	2.56 (2.20–2.97)	<0.001
**Co-morbidity**						
**Diabetic** **	52	*6*	206	*5*	1.28 (0.94–1.76)	0.118
**Habits**						
**Smoker** **	396	*46*	1623	*37*	1.41 (1.22–1.64)	<0.001
**Alcohol use** **	250	*29*	1315	*30*	0.93 (0.79–1.09)	0.415

*Details not available for 55 patients in the elderly group and 222 in the younger.

**Details not available for 2 patients in the younger group.

$ Odds ratio (95% CI) calculated with younger group as reference using chi-square test.

### TB symptoms and diagnosis (Table 2)

Pulmonary TB disease (96% vs. 91%; p<0.001) and eligibility for Category III ie.smear negative and non-severe extra-pulmonary TB (41% vs. 33%; p<0.001) were higher among the elderly (Table 2)

**Table 2:** TB symptoms, diagnosis and treatment in the elderly and younger group.

Characteristics		Elderly n = 865		Younger n = 4343		OR (95% CI)$	P value
		n	%	n	%		
**Type of disease**	Pulmonary	834	96	3943	91	2.73 (1.88–3.96)	<0.001
**Case detection**	Survey (Active)	168	19	481	11	1.83 (1.49–2.23)	<0.001
**Anti-TB treatment**							
New patients	Category I	397	46	2205	51	0.82 (0.71–0.95)	0.01
	Category III	356	41	1447	33	1.39 (1.20–1.63)	<0.001
Previously treated	Category II	112	13	691	16	0.79 (0.63–0.98)	0.03
**Pulmonary TB**		**n = 834**		**n = 3943**			
**Symptoms (Irrespective of duration)**							
	Cough*	700	84	3290	83	1.04 (0.8–1.3)	0.758
	Breathlessness@	336	40	1421	36	1.4 (1.2–1.6)	0.021
	Chest pain*	520	62	2569	65	0.9 (0.8–1.04)	0.130
	Hemoptysis*	198	24	1115	28	0.8 (0.7–0.9)	0.008
**First action for relief from respiratory complaints** *	Govt	419	50	1829	46	1.2 (1.0–1.4)	0.043
Private		289	35	1600	41	0.8 (0.7–0.9)	0.002
Home/Pharmacy		120	14	503	13	1.1 (0.9–1.4)	0.204
Alternate Medicine		6	1	11	<1	2.6 (0.8–7.7)	0.052
**Anti-TB treatment**							
New patients	Category I	389	47	2117	54	0.7 (0.6–0.9)	<0.001
	Category III	333	40	1143	29	1.6 (1.4–1.9)	<0.001
Previously treated	Category II	112	13	683	17	0.7 (0.6–0.9)	0.006
**Sputum smear**	Negative	385	46	1408	36	1.54 (1.3–1.8)	<0.001

*Details not available for 2 patients in the younger group.

@ Details not available for 223 patients in the elderly and 1114 patients in the younger group.

$ Odds ratio (95% CI) calculated with younger group as reference using chi-square test.

Of the 834 elderly and 3943 younger pulmonary TB patients, 109 (13%) and 488 (12%) were asymptomatic respectively (OR, 1.06; 95%CI, 0.85–1.34); p = 0.582. Among the symptomatics, a higher proportion of elderly TB patients (40% vs. 36%; p = 0.021) reported breathlessness, while cough was similar between the two groups (84% vs. 83%). (Table 2) The RNTCP definition of a TB suspect during the period of this study was cough of more than 3 weeks duration. Among the 700 elderly and 3290 younger pulmonary TB patients who reported cough, duration of ≥21 days was reported by 627 (89%) of elderly and 2922 (89%) younger patients; p = 0.56. Among the 336 elderly and 1421 younger patients with breathlessness, duration of ≥21 days duration was reported by 296 (88%) of elderly and 1195 (84%) younger patients; p = 0.07. There was no difference in the duration of symptoms (≥21 days) prior to diagnosis, between the elderly and younger TB patients

Elderly patients were less likely to contact a private provider as their first action for relief from respiratory complaints (35% vs. 41%; p = 0.002). (Table 2) The median duration between onset of symptoms and the first action was 2 weeks (IQR: 1–4) and did not differ between the elderly and the younger group. Initial sputum smear negativity (46% vs. 36%; p<0.001) was higher in the elderly with pulmonary TB.

All the 649 patients identified by active case finding during a prevalence survey had pulmonary TB. The proportion with asymptomatic disease was higher among those identified through active case finding (15% vs. 12%; p = 0.021) compared to passive case finding. Breathlessness (53% vs. 35%; p<0.001) and hemoptysis (28% vs. 23%; p<0.01) were higher among those identified by passive case finding while proportion of those with cough was similar (83% vs. 85%; p = 0.43) in active and passive case finding. The proportion of patients with symptoms of ≥21 days duration was higher in the group identified by active compared to passive case finding; cough (92% vs. 88%; p<0.01), breathlessness (90% vs. 84%; p<0.001).Smear negativity rate (43% vs. 37%; p<0.001) was higher among those identified by active case finding compared to passive case finding.

Active case finding identified a higher proportion of elderly with TB (19% vs. 11%; p<0.001). (Table 2) There were no significant differences between the elderly and younger with respect to symptoms and their duration in patients detected by active case finding. The sputum smear negativity rate was similar between the two groups in active case finding, however sputum smear negativity rate was higher in elderly in passive case finding (46% vs. 35%; p<0.001).

### Drug resistance, smear conversion and treatment outcomes in newly diagnosed TB patients

#### Category I

There were 397 elderly and 2205 younger patients who were treated with the Category I regimen. Overall elderly patients had less favorable outcome i.e cured/treatment completed (71% vs. 80%; p<0.001) with more defaulters (14% vs. 11%; p = 0.09) and deaths (10% vs. 4%; p<0.001).(Table 3) Data on drug-related problems, due to the bulk of drugs or side effects was available for 289 elderly and 1648 younger TB patients. Elderly 183 (63%) reported more problems compared to the younger 897 (54%); (OR, 1.44; 95%CI, 1.12–1.87); p = 0.005.

**Table 3:** Drug susceptibility profile, Smear conversion and TB Treatment outcomes in newly diagnosed TB patients in the elderly and younger group.

	Elderly		Younger		OR (95% CI)$	P value
**Category I**	**n = 397**		**n = 2205**			
**Overall treatment outcome**	**n**	**%**	**n**	**%**		
Cured/Treatment Completed	283	*71*	1770	*80*	0.61 (0.48–0.78)	<0.001
Failure	17	*4*	97	*4*	1.97 (0.54–1.66)	0.92
Loss to follow-up	56	*14*	247	*11*	1.30 (0.90–1.80)	0.09
Death	38	*10*	86	*4*	2.60 (1.70–3.90)	<0.001
Transferred	3	*1*	5	*<1*	3.75 (0.89–15.79)	0.07
**Smear positive PTB**	**n = 352**		**n = 1933**			
**Smear conversion at the end of Intensive phase**	**n**	**%**	**n**	**%**		
Yes	272	77	1651	85	0.58 (0.44–0.77)	<0.001
**Culture result available**	**n = 336**		**n = 1856**			
Positive	294	*88*	1602	*86*	1.20 (0.80–1.60)	0.55
**Drug susceptibility available**	**n = 293**		**n = 1597**			
Sensitive	256	*87*	1349	*84*	1.27 (0.87–1.90)	0.20
Any resistance						
H	21	*7*	169	*11*	0.65 (0.40–1.10)	0.08
SM	21	*7*	141	*9*	0.79 (0.47–1.29)	0.35
R	4	*1*	30	*2*	0.72 (0.18–2.07)	0.54
HR	3	*1*	25	*2*	0.65 (0.12–2.15)	0.48
**Treatment outcome of smear positive PTB treated with Category I**						
Cured/Treatment completed	248	*70*	1546	*80*	0.60 (0.46–0.78)	<0.001
Failure	16	*5*	96	*5*	0.91(0.49–1.58)	0.74
Loss to follow-up	54	*15*	219	*11*	1.40 (1.0–1.97)	0.03
Death	31	*9*	68	*4*	2.60 (1.6–4.20)	<0.001
Transferred	3	*1*	4	*<1*	4.67 (1.04–21.01)	0.04
**Category III**	**n = 356**		**n = 1447**			
**Treatment outcome**	**n**	**%**	**n**	**%**		
Treatment Completed	275	*77*	1238	*85*	0.57 (0.43–0.77)	<0.001
Failure	6	*2*	19	*1*	1.30 (0.42–3.39)	0.59
Loss to follow-up	56	*16*	142	*10*	1.70 (1.20–2.40)	0.001
Death	19	*5*	45	*3*	1.81 (0.96–3.11)	0.04
Transferred	0	*0*	3	*<1*	NA	**-**

$ Odds ratio (95% CI) calculated with younger group as reference using chi-square test.

Among smear positive patients (352 elderly and 1933 younger), lower smear conversion rate at the end of IP (77% vs. 85%; p<0.001) and higher loss to follow-up (15% vs. 11%; p = 0.03) and death rates (9% vs. 4%; p<0.001) were observed in the elderly. (Table 3)

Drug susceptibility results were available for the 293 elderly and 1597 younger patients. Similar proportions of patients had *Mycobacterium tuberculosis (M.tb)* susceptible to all first line anti-TB drugs (elderly 87% vs. younger 84%) with no difference in MDR-TB rates. (Table 3)

#### Category III

There were 356 elderly and 1447 younger patients who were treated with the Category III regimen. Elderly patients were less likely to complete treatment (77% vs. 85%; p<0,001) and more likely to be lost to follow-up (16% vs. 10%; p = 0.001). (Table 3)

Among 252 elderly and 1014 younger TB patients with data available, elderly 95 (38%) reported less drug related problems compared to the younger group 532 (52%); (OR, 0.55; 95%CI, 0.41–0.73); p<0.0001.

### Drug resistance, smear conversion and TB Treatment outcomes in previously treated TB patients

There were 112 elderly and 691 younger patients who were treated with the Category II regimen. Treatment outcomes did not differ between the groups. (Table 4) Drug related problems were similar; (elderly 38 (48%) vs. younger 261 (55%); (OR, 0.73; 95%CI, 0.46–1.18); p = 0.20 among 80 elderly and 473 younger patients for whom data was available.

**Table 4:** Drug susceptibility profile, Smear conversion and TB Treatment outcomes in previously treated TB patients in the elderly and younger group.

	Elderly		Younger		OR (95% CI)$	P value
**Category II**	**n = 112**		**n = 691**			
**Overall treatment outcome**	**n**	**%**	**n**	**%**		
Cured/Treatment Completed	54	48	316	46	1.10 (0.72–1.68)	0.62
Failure	4	4	57	8	0.41 (0.14–1.18)	0.10
Loss to follow-up	40	36	264	38	0.90 (0.60–1.40)	0.61
Death	11	10	47	7	1.50 (0.70–3.00)	0.25
Transferred	3	3	7	1	2.51 (0.63–9.99)	0.19
**Smear positive PTB**	**n = 97**		**n = 602**			
**Smear conversion at the end of Intensive phase**	**n**	**%**	**n**	**%**		
Yes	65	67	362	60	1.35 (0.86–2.12)	0.20
**Culture result available**	**n = 92**		**n = 552**			
Positive	67	73	453	82	0.60 (0.34–1.02)	0.04
**Drug susceptibility available**	**n = 66**		**n = 452**			
Sensitive	42	64	263	58	1.26 (0.72–2.25)	0.40
Any resistance						
H	20	30	167	37	0.75 (0.42–1.32)	0.31
SM	8	12	74	16	0.67 (0.30–1.49)	0.33
R	4	6	54	12	0.46 (0.16–1.34)	0.16
HR	4	6	51	11	0.49 (0.17–1.42)	0.19
**Treatment outcome of smear positive PTB treated with Category II**						
Cured/Treatment completed	46	48	256	43	1.21 (0.77–1.91)	0.37
Failure	4	4	56	9	0.42 (0.11–1.18)	0.09
Loss to follow-up	36	37	245	41	0.90 (0.50–1.40)	0.50
Death	9	9	38	6	1.60 (0.60–3.30)	0.28
Transferred	2	2	7	1	1.59 (0.32–7.87)	0.57

$ Odds ratio (95% CI) calculated with younger group as reference using chi-square test.

Sputum smear conversion rate at the end of IP in the elderly was 67% compared to 60% in younger; p = 0.20). Treatment outcomes did not differ between the two groups. (Table 4). Drug susceptibility profile was available for 66 elderly and 452 younger patients. Susceptibility to first line anti-TB drugs did not differ between the groups. MDR-TB rates were lower among the elderly than in the younger group, but this was not statistically significant (6% vs. 11%, p = 0.19). (Table 4)

## Discussion

TB is emerging as a significant health problem in the elderly [17] and the control of TB in this group is essential for the overall success of TB control programmes. This study has documented that elderly TB patients are less likely to have smear positive disease and newly diagnosed elderly TB patients are more likely to default or die and report drug side effects, which is a matter of concern.

Sputum smear microscopy is the cornerstone of TB diagnosis. Similar to previous studies [9], [12], [18], we observed higher initial sputum smear negativity in the elderly. In support of this, a higher proportion of elderly patients received the Category III regimen compared to the younger (41% vs. 33%). In the RNTCP, diagnosis of smear negative TB is based on the persistence of respiratory symptoms and/or radiological abnormality despite a course of antibiotics. We do not have data on the proportion of patients in this study with radiological abnormality. Sputum culture is not routinely done in RNTCP and therefore we do not know the proportion of smear negative patients who were culture positive. Currently the TB Control programme classifies TB patients based on history of previous treatment as “New” or “previously treated” and offers only 2 types of regimen - Category I and II [19]. Nevertheless, the higher initial sputum smear negativity observed in the elderly may delay the diagnosis of TB. TB diagnostic algorithms should include other diagnostic tools apart from smear microscopy (eg. Xpert MTB/RIF) while evaluating the elderly for diagnosis of TB.

Smear conversion at the end of IP is an early predictor of treatment success [20]. Elderly new smear positive TB patients had lower smear conversion rates at the end of IP in this study (77% vs. 85%). This is similar to a previous study in which the sputum conversion rates at the end of the intensive phase were found to be significantly lower in the elderly in comparison to the younger TB patients (75.3% vs. 85.7%: p<0.01) [8]. There could be many reasons for this; including modification of treatment due to adverse effects of anti-TB drugs and associated illness [8]. The possibility of malabsorption of anti-TB drugs in the elderly could also be considered since malabsorption of calcium, folate and Vitamin B_12_ has been reported in the elderly [21].

The cure/treatment completion rate among new elderly TB patients was observed to be 71% in our study which is lower than the RNTCP objective of 85% cure rate [13]. This can be attributed to higher death (10%) and loss to follow-up rates (14%). A recent study from South India has documented a significantly higher risk (RR-1.4, 95% CI 1.2–1.6) of unfavourable treatment outcomes in older TB patients (16% compared with 11% for all others) with a stronger association among new TB patients [7]. Death rate of 8% among new smear positive elderly TB patients has been documented in previous studies [12], [22]. The higher death rate among elderly TB patients has to be interpreted with caution since mortality increases with age. Higher mortality among TB patients aged 70 years or more has been reported [7]. The cause of death in elderly TB patients can be difficult to determine, as TB may not be the only life-threatening disease and autopsies are rarely performed [23]. Nevertheless, in a study from Germany, TB as a post mortem diagnosis was more frequently established among elderly patients [18].

High loss to follow-up rates of 10.7% and 14.2% in the elderly were observed in previous studies [8], [12] similar to our observation of 14%. The reasons cited for loss to follow-up in the elderly are poor memory, poor eyesight, low tolerance to therapy, mental confusion, associated depression, concomitant illness and logistic problems for regular visits to treatment centre [8], [17]. Loss to follow-up rate in the elderly could be reduced by ensuring good quality geriatric health services at the primary care level to address the various needs of the elderly population. Community geriatric health workers to provide home care may contribute to better TB management in the elderly.

We observed similar treatment outcomes among previously treated elderly and younger TB patients. Loss to follow-up rate was greater than 35% in these patients. Higher loss to follow-up rates for re-treatment patients have been documented in previous studies [12], [24]. Adverse effects to anti-TB drugs either due to the bulk or toxicity was higher in elderly new TB patients (63% vs. 54%) in this study. A study from Korea documented higher drug-induced hepatitis, cutaneous toxicity, neurotoxicity, gastrointestinal disturbances, arthralgia, and flu-like syndrome in the elderly [25]. Advancing age as an important predictor of hepatotoxicity due to H and R has been documented [26], [27]. Polypharmacy and age-related changes in responses to drugs and their elimination due to compromised hepatic and renal functions, contribute to drug adverse effects [26]. Health care workers need to be aware of potential drug adverse effects for prompt intervention and appropriate management.

*M.tb* drug resistance rates were marginally lower, though not statistically significant, among elderly compared to younger patients, both among new and previously treated patients in this study. This is similar to a previous study which documented no significant differences in isoniazid-resistant TB and MDR TB between the two age groups [28]. Analysis of TB notification data from Germany documented markedly lower drug resistance rates in the elderly for any drug resistance (6.5% vs 13.9%; p<0.001) and for MDR (0.6% vs. 3.1%; p<0.001) [18]. The reason for this phenomenon could be that TB disease in the elderly is largely due to re-activation of infection acquired in the distant past when drug resistant rates were lower.

Pulmonary TB (PTB) was common in the elderly in our study. This is in conformity with previous studies which documented higher prevalence of PTB in the elderly [8], [12]. Pulmonary TB is a disease with male preponderance. Higher proportion of males in the elderly observed in our study is similar to previous reports [8], [9].

Smoking is a risk factor for the development of TB [29]. A higher proportion of elderly males (46%vs 37%) were smokers in this study. A study from Amritsar among hospitalised TB patients showed no difference in smoking between the elderly and younger [10] while a descriptive study in a cohort of 100 patients aged >50 yrs from Himachal Pradesh documented 68% smoking [11]. Smoking cessation interventions focusing on the elderly will be important to reduce the burden of complications due to TB.

Associated co-morbidities in the elderly are a challenge to both TB diagnosis and treatment. We observed similar prevalence of diabetes mellitus in both age groups. Prevalence of co-morbidities namely cardiovascular diseases, diabetes mellitus, and airway obstructive disease has been reported to be higher in older TB patients [28].

A systematic review documented old age as a risk factor for TB diagnostic delay, with factors such as poverty, social isolation, and specific health-seeking behaviour hampering the elderly in gaining timely access to medical care [30]. We observed 63% illiteracy and a higher case detection rate by active case finding (through survey) in the elderly. In addition, the elderly were economically dependent, as 58% were unemployed which implies that family support was necessary for medical help. Organizing frequent TB screening camps and other methods for active case finding, mobile clinics and increasing awareness through mass media may enhance early TB case detection in the elderly.

The limitations of our study include the possibility of potential misclassification of some Category II as Category I patients due to concealed history of previous anti-TB treatment at the time of diagnosis. The information on some important risk factors, such as HIV infection that could influence treatment outcomes was not available. Diabetes was self reported and this probably contributed to its lower prevalence. In addition, we were unable to collect more details pertaining to toxicity of anti-TB treatment and investigate the cause of death. Data for radiological extent of disease and the exact duration of symptoms prior to diagnosis were not available.

In summary, our study has shown that elderly TB patients are less likely to have smear positive disease though the majority had pulmonary TB. Newly diagnosed elderly TB patients are more likely to be lost to follow-up or die and report drug side effects. Programme managers should develop appropriate interventions in consultation with geriatric specialists, for effective management of TB in this vulnerable population.

## References

[1] Ingle GK, Nath A (2008) Geriatric health in India: concerns and solutions. Indian J Community Med 33 (4) 214–218. 19876492 10.4103/0970-0218.43225 PMC2763704

[2] Situational analysis of elderly in India. Central Statistics Office Ministry of Statistics & Programme Implementation Government of India, 2011. http://mospi.nic.in/mospi_new/upload/elderly_in_india.pdf. Accessed 2013 April 21.

[3] Rath SP, Das B, Mishra SK (2011) Demographic dynamics of India's population - A reference study of census-2011 with backdrops and future trends. Int J of Business and Management tomorrow 1: 1–22 (http://www.ijbmt.com/issue/90.pdf). Accessed 2013 April 21.

[4] WHO.Global tuberculosis report 2013. http://apps.who.int/iris/bitstream/10665/91355/1/9789241564656_eng.pdf Accessed 2013 Dec 7

[5] Stead WW (1965) The pathogenesis of pulmonary tuberculosis among older persons. Am Rev Respir Dis 91: 811–822. 14294702 10.1164/arrd.1965.91.6.811

[6] Schaaf HS, Collins A, Bekker A, Davies PD (2010) Tuberculosis at extremes of age. Respirology 15 (5) 747–763. 20546192 10.1111/j.1440-1843.2010.01784.x

[7] Ananthakrishnan R, Kumar K, Ganesh M, Kumar AMV, Krishnan N, et al (2013) The Profile and Treatment Outcomes of the Older (Aged 60 Years and Above) Tuberculosis Patients in Tamilnadu, South India. PLoS ONE 8 (7) e67288 doi:10.1371/journal.pone.0067288 23861755 10.1371/journal.pone.0067288 PMC3704605

[8] Arora VK, Singla N, Sarin R (2003) Profile of geriatric patients under DOTS in Revised National Tuberculosis Control Programme. Indian J Chest Dis Allied Sci 45 (4) 231–235. 12962456

[9] Gaur SN, Dhingra VK, Rajpal S, Aggarwal JK (2004) Meghna (2004) Tuberculosis in the elderly and their treatment outcome under DOTS. Indian J Tuberc 51: 83–87.

[10] Bhushan B, Kajal NC, Maske A, Singh SP (2012) Manifestations of tuberculosis in elderly versus young hospitalised patients in Amritsar, India. Int J Tuberc Lung Dis 16 (9) 1210–1213. 22794086 10.5588/ijtld.11.0778

[11] Arora VK, Bedi RS (1989) Geriatric Tuberculosis in Himachal Pradesh: A Clinical Radiological Profile. J Assoc Physicians India 37: 205–207. 2768162

[12] Pardeshi G, Deshmukh D (2007) Disease characteristics and treatment outcomes in elderly tuberculosis patients on DOTS. Ind J Comm Med 32: 292–294.

[13] Central TB Division (CTD), Directorate General of Health Services, Ministry of Health and Family Welfare, Government of India. Managing Revised National TB Control Programme in your area – A training course. Dated April 2005. Module 4 – Administering treatment. Pg: 77–114; New Delhi: CTD.

[14] Allen B, Baker FJ (1968) Mycobacteria: isolation, identification and sensitivity testing. London, UK: Butterworth.

[15] Canetti G, Fox W, Khomenko A, Mahler HT, Menon NK, et al (1969) Advances in technique of testing mycobacterial drug sensitivity, and the use of sensitivity tests in tuberculosis control programmes. Bull World Health Organ 41: 21–43. 5309084 PMC2427409

[16] Tuberculosis Research Centre, Madras (1983) Study of chemotherapy regimens of 5 and 7 months' duration and the role of corticosteroids in the treatment of sputum positive patients with pulmonary tuberculosis in south India. Tubercle 64: 73–91. 6351390 10.1016/0041-3879(83)90032-6

[17] Sood R (2000) The problem of geriatric tuberculosis. J Acad Clin Med 5: 156–162.

[18] Hauer B, Brodhun B, Altmann D, Fiebig L, Loddenkemper R, et al (2011) Tuberculosis in the elderly in Germany. Eur Respir J 38 (2) 467–470. 21804163 10.1183/09031936.00199910

[19] Central TB Division (CTD), Directorate General of Health Services, Ministry of Health and Family Welfare, Government of India. Revised National Tuberculosis Control Programme (RNTCP).Training Module for Medical Practitioners. Dated December 2010. Chapter 3 – Administering treatment. Pg: 27–30; New Delhi: CTD.

[20] Zhao FZ, Levy MH, Wen S (1997) Sputum microscopy results at two and three months predict outcome of tuberculosis treatment. Int J Tuberc Lung Dis 1 (6) 570–572. 9487456

[21] Holt PR (2001) Diarrhea and malabsorption in the elderly. Gastroenterol Clin North Am 2001 30 (2) 427–444. 10.1016/s0889-8553(05)70189-8 11432299

[22] Rawat J, Sindhwani G, Juyal R (2008) Clinico-radiological profile of new smear positive pulmonary tuberculosis cases among young adult and elderly people in a tertiary care hospital at Deheradun (Uttarakhand). Indian J Tuberc 55 (2) 84–90. 18516824

[23] Brinkmann B, Du Chesne A, Vennemann B (2002) Recent data for frequency of autopsy in Germany. [Article in German] Dtsch Med Wochenschr 127 (15) 791–795. 11951136 10.1055/s-2002-25021

[24] Santha T, Garg R, Frieden TR, Chandrasekaran V, Subramani R, et al (2002) Risk factors associated with default, failure and death among tuberculosis patients treated in a DOTS programme in Tiruvallur District, South India, 2000. Int J Tuberc Lung Dis 6 (9) 780–788. 12234133

[25] Lee J H, Han DH, Song JW, Chung HS (2005) Diagnostic and therapeutic problems of pulmonary tuberculosis in elderly patients. J Korean Med Sci 20: 784–789. 16224152 10.3346/jkms.2005.20.5.784 PMC2779275

[26] Teale C, Goldman JM, Pearson SB (1993) The association of age with the presentation and outcome of tuberculosis: a five-year survey. Age Ageing 22 (4) 289–293. 8213336 10.1093/ageing/22.4.289

[27] Pande JN, Singh SP, Khilnani GC, Khilnani S, Tandon RK (1996) Risk factors for hepatotoxicity from antituberculosis drugs: a case-control study. Thorax 51 (2) 132–136. 8711642 10.1136/thx.51.2.132 PMC473016

[28] Kwon YS, Chi SY, Oh IJ, Kim KS, Kim YI, et al (2013) Clinical characteristics and treatment outcomes of tuberculosis in the elderly: a case control study. BMC Infect Dis 13: 121. 23510403 10.1186/1471-2334-13-121 PMC3599150

[29] Kolappan C, Gopi PG (2002) Tobacco smoking and pulmonary tuberculosis. Thorax 57: 964–966. 12403879 10.1136/thorax.57.11.964 PMC1746222

[30] Storla DG, Yimer S, Bjune GA (2008) A systematic review of delay in the diagnosis and treatment of tuberculosis. BMC Public Health 8: 15. 18194573 10.1186/1471-2458-8-15 PMC2265684

